# Supplementary material for: Cancer-associated fibroblast-secreted CXCL16 attracts monocytes to promote stroma activation in triple-negative breast cancers
Source: Nat Commun. 2016 Oct 11;7:13050. doi: 10.1038/ncomms13050 (PMC5062608; doi:10.1038/ncomms13050)
Supplement: Supplementary Information — Supplementary Figures 1-7, Supplementary Tables 1-5 and Supplementary References [file ncomms13050-s1.pdf]

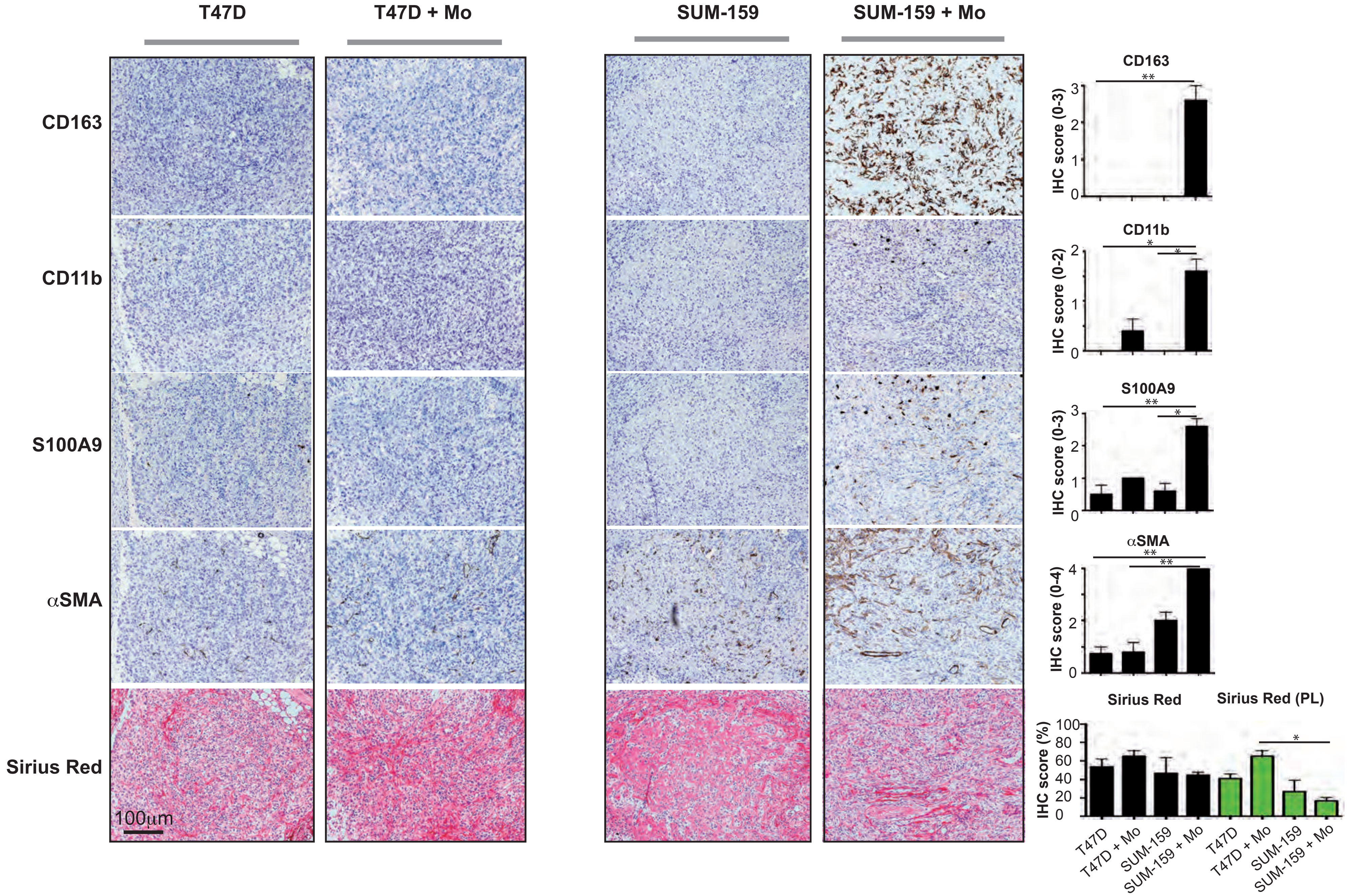
T47D
T47D + Mo
SUM-159
SUM-159 + Mo
CD163
CD11b
S100A9
αSMA
Sirius Red
100μm
CD163
IHC score (0-3)
CD11b
IHC score (0-2)
S100A9
IHC score (0-3)
αSMA
IHC score (0-4)
Sirius Red
Sirius Red (PL)
IHC score (%)
T47D
T47D + Mo
SUM-159
SUM-159 + Mo
T47D
T47D + Mo
SUM-159
SUM-159 + Mo

Supplementary Figure 1. *Immunohistochemistry of xenografts*

Tumor xenografts consisting of triple negative (TN) SUM-159 breast cancer cells co-transplanted with primary human monocytes, express more myeloid-related, immunosuppressive and activated fibroblast markers than luminal A T47D / monocyte xenografts. The xenografts were grown in highly immunodeficient NSG-mice (see Material and methods), and sections from the tumors were stained with myeloid (CD163, CD11b, S100A9) and the activated fibroblast marker αSMA. The two cell lines chosen are negative for S100A9 [1]. Immunohistochemistry was performed using the indicated antibodies. All histological sections were counterstained with HE. N=5 mice were analyzed for each group; Grafts were analyzed on day 21. The histograms to the right show the mean value for each IHC score with statistical analysis. IHC scores are shown in Supplementary Table 2. *=$p<0.05$, **=$p<0.01$ ANOVA non-parametric Kruskal Wallis test. N=5. Error bars indicate SEM.

Fig 2

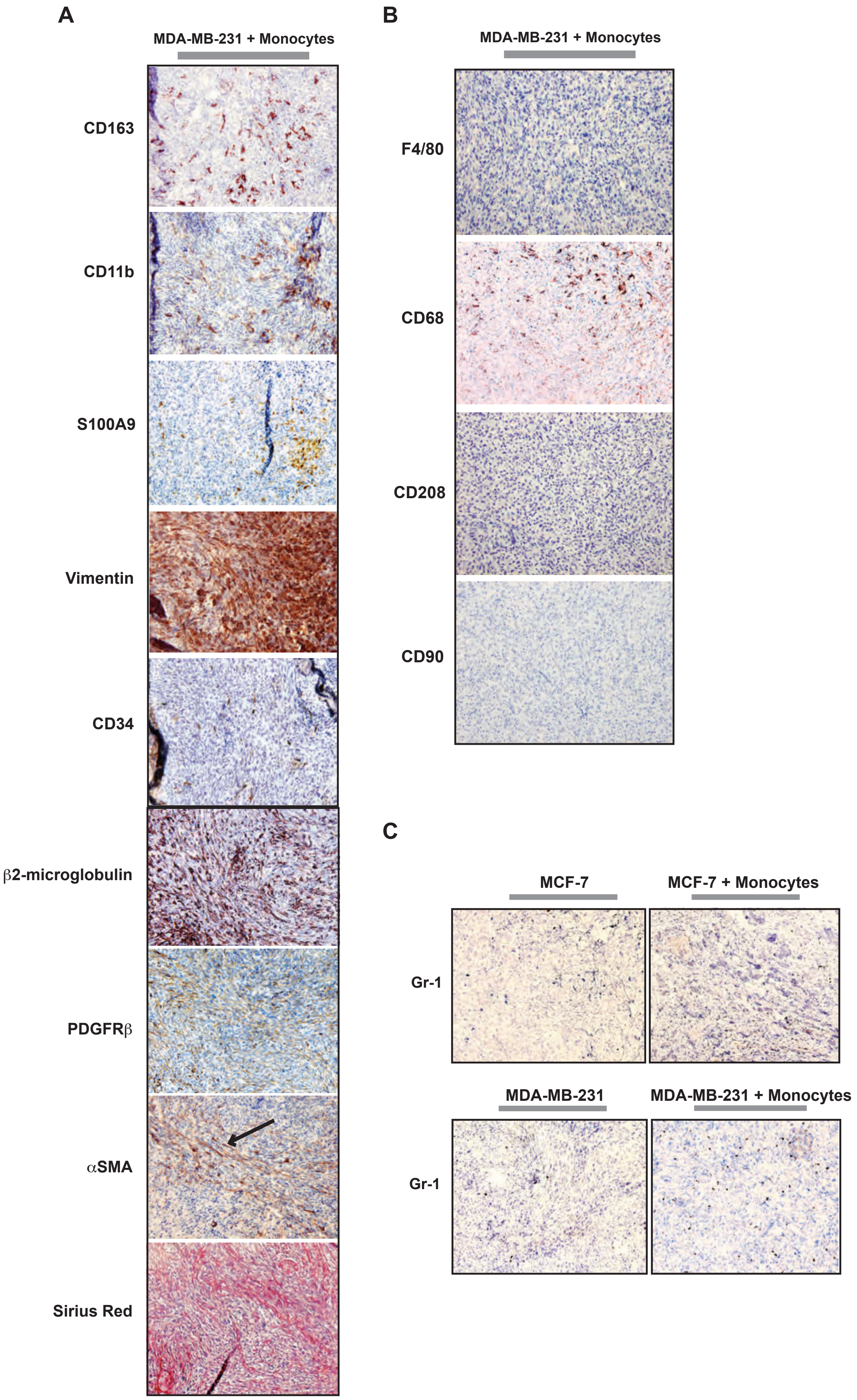
A
MDA-MB-231 + Monocytes
CD163
CD11b
S100A9
Vimentin
CD34
β2-microglobulin
PDGFRβ
αSMA
Sirius Red
B
MDA-MB-231 + Monocytes
F4/80
CD68
CD208
CD90
C
MCF-7
MCF-7 + Monocytes
Gr-1
MDA-MB-231
MDA-MB-231 + Monocytes
Gr-1

Supplementary Figure 2. *Immunohistochemistry of xenografts*

(A) Xenografts consisting of triple-negative (TN) MDA-MB-231 breast cancer cells co-transplanted with primary human monocytes, into NSG mice. One graft representing a *low* myeloid cell take is shown for the TN MDA-MB-231 / monocyte co-transplant group.

(B) Xenografts consisting of triple-negative (TN) MDA-MB-231 breast cancer cells co-transplanted with primary human monocytes, into NSG mice and stained for murine macrophages (F4/80), human macrophages (CD68) human myeloid dendritic cells (mDCs; CD208) and human fibroblasts (CD90).

(C) Xenografts consisting of luminal A (MCF-7) and triple-negative (TN) MDA-MB-231 breast cancer cells transplanted with or without primary human monocytes, into NSG mice and stained for the mouse myeloid marker, Gr1. All histological sections were counterstained with HE. N=5 for each group.

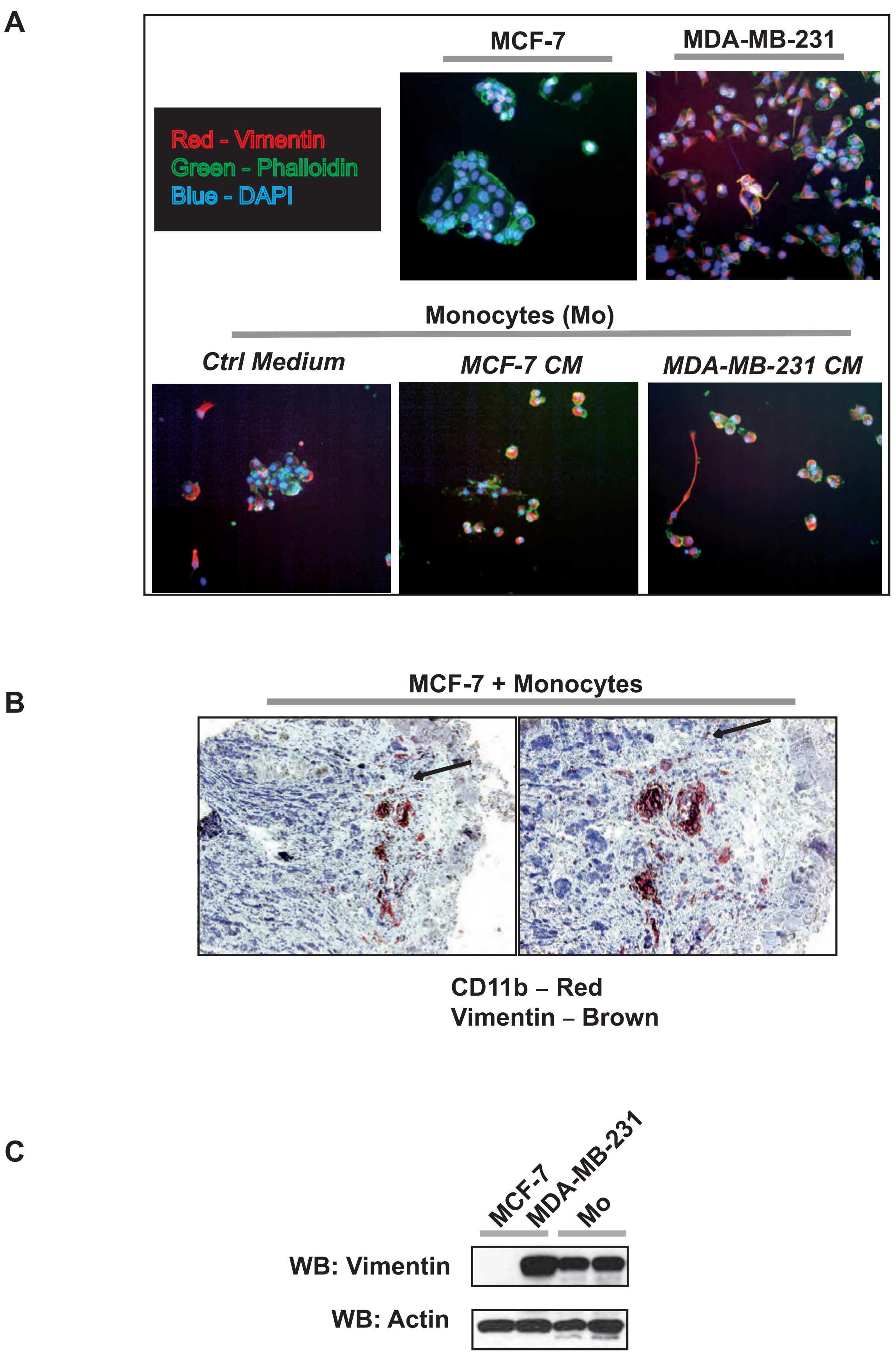
A
MCF-7
MDA-MB-231
Red - Vimentin
Green - Phalloidin
Blue - DAPI
Monocytes (Mo)
Ctrl Medium
MCF-7 CM
MDA-MB-231 CM
B
MCF-7 + Monocytes
CD11b – Red
Vimentin – Brown
C
MCF-7
MDA-MB-231
Mo
WB: Vimentin
WB: Actin

Supplementary Figure 3. *Vimentin is expressed by myeloid cells*

(A) Immunofluorescence of primary human monocytes cultured with breast cancer cell conditioned medium or under control conditions (only GM-CSF) and stained for Vimentin (red), phalloidin (to stain actin filaments; green) and DAPI (nuclear stain; blue). MCF-7 and MDA-MB-231 breast cancer cells were used as negative and positive controls, respectively.

(B) Double staining IHC of CD11b and vimentin in xeno-transplants from MCF-7 / monocytes tumors as indicated. Black arrows show staining with vimentin but not CD11b.

(C) Western blot (WB) of vimentin expression in MCF-7 and MDA-MB-231 breast cancer cells or human primary monocytes (Mo) isolated from samples from two healthy blood donors. Actin is used as a loading control.

Fig 4

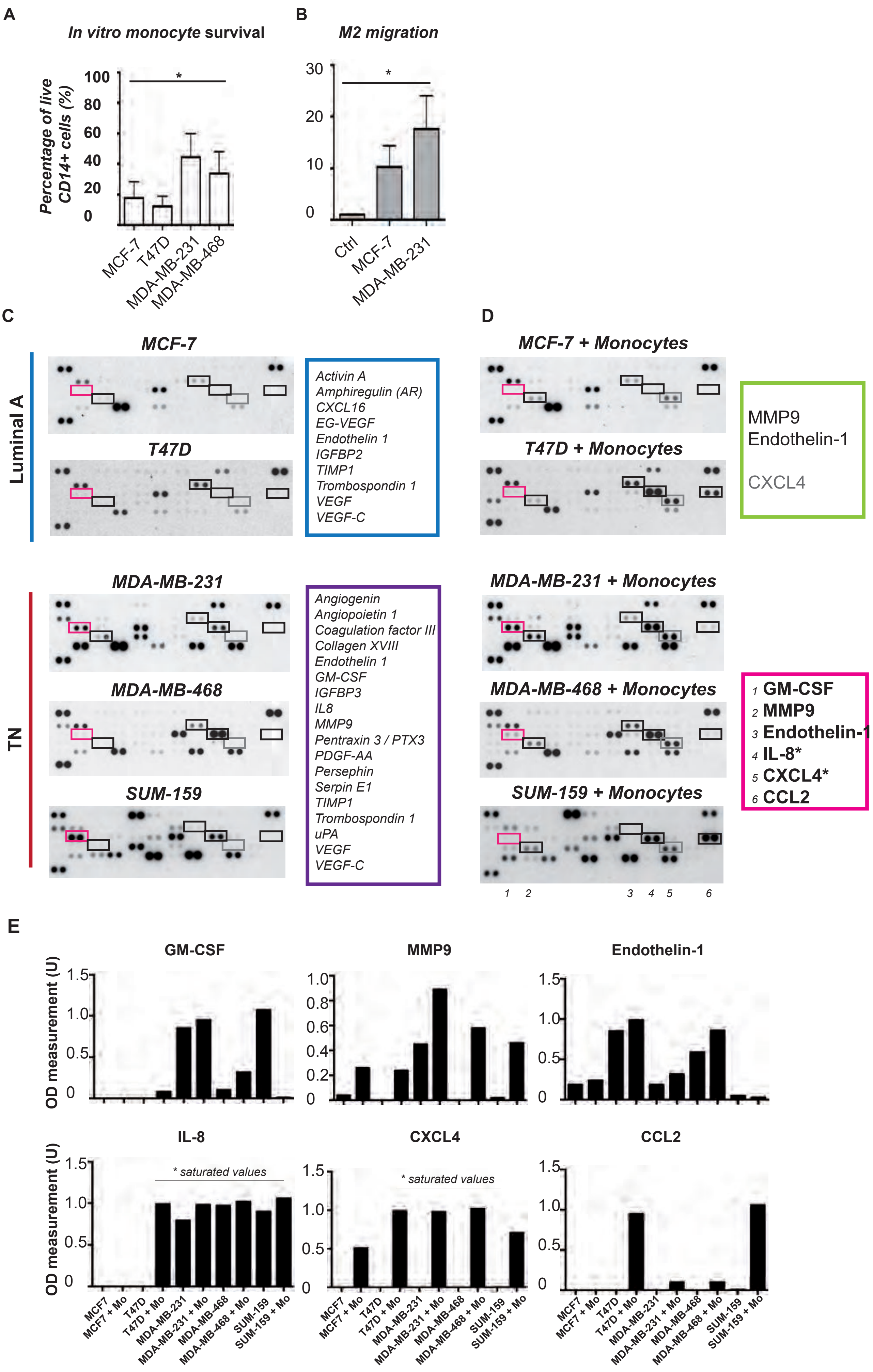

A
In vitro monocyte survival
Percentage of live CD14+ cells (%)
MCF-7
T47D
MDA-MB-231
MDA-MB-468
B
M2 migration
Ctrl
MCF-7
MDA-MB-231
C
MCF-7
T47D
Luminal A
Activin A
Amphiregulin (AR)
CXCL16
EG-VEGF
Endothelin 1
IGFBP2
TIMP1
Trombospondin 1
VEGF
VEGF-C
MDA-MB-231
MDA-MB-468
SUM-159
TN
Angiogenin
Angiopoietin 1
Coagulation factor III
Collagen XVIII
Endothelin 1
GM-CSF
IGFBP3
IL8
MMP9
Pentraxin 3 / PTX3
PDGF-AA
Persephin
Serpin E1
TIMP1
Trombospondin 1
uPA
VEGF
VEGF-C
D
MCF-7 + Monocytes
T47D + Monocytes
MMP9
Endothelin-1
CXCL4
MDA-MB-231 + Monocytes
MDA-MB-468 + Monocytes
SUM-159 + Monocytes
1 GM-CSF
2 MMP9
3 Endothelin-1
4 IL-8*
5 CXCL4*
6 CCL2
E
GM-CSF
MMP9
Endothelin-1
IL-8
CXCL4
CCL2
OD measurement (U)
* saturated values
MCF7
MCF7 + Mo
T47D
T47D + Mo
MDA-MB-231
MDA-MB-231 + Mo
MDA-MB-468
MDA-MB-468 + Mo
SUM-159
SUM-159 + Mo

Supplementary Figure 4. *Effect of breast cancer cells on monocytes*

(A) Survival of isolated human primary monocytes in breast cancer cell conditioned medium, grown for 7 days, was assessed. 7AAD and $CD14^{+}$staining was performed to analyze the content of live monocyte/macrophages in each culture. *=p<0.05 ***=p<0.001. ANOVA. N=5. Error bars indicate SEM.

(B) Boyden chamber migration assay of primary human M2 macrophages migrating towards control medium or breast cancer cell conditioned medium. *=p<0.05 ANOVA. N=8. Error bars indicate SEM.

(C) Human angiogenesis array proteome profiler of supernatants from luminal A (MCF-7 and T47D) or triple negative (TN) (MDA-MB-231, MDA-MB-468 and SUM-159) breast cancer cells before monocyte co-culture. The factors in the blue box are expressed typically in luminal A breast cancer cells, and the factors in the purple box are expressed typically in TN breast cancer cells.

(D) Human angiogenesis array proteome profiler of supernatants from co-cultures of human primary monocytes and luminal A (MCF-7 and T47D) or TN (MDA-MB-231, MDA-MB-468 and SUM-159) breast cancer cells. The factors in the green and pink boxes are specifically upregulated upon co-culture with monocytes, with the criteria if upregulated in both cultures of luminal A or TN breast cancer/monocyte co-cultures, respectively. The star (*) indicates saturated values. The numbers (1-7) below the boxes indicate corresponding dots with each factor.

(E) The histograms represent the OD values for each factor in relation to the reference dots A1-2 (upper left corner) for each filter. N=2.

Fig 5

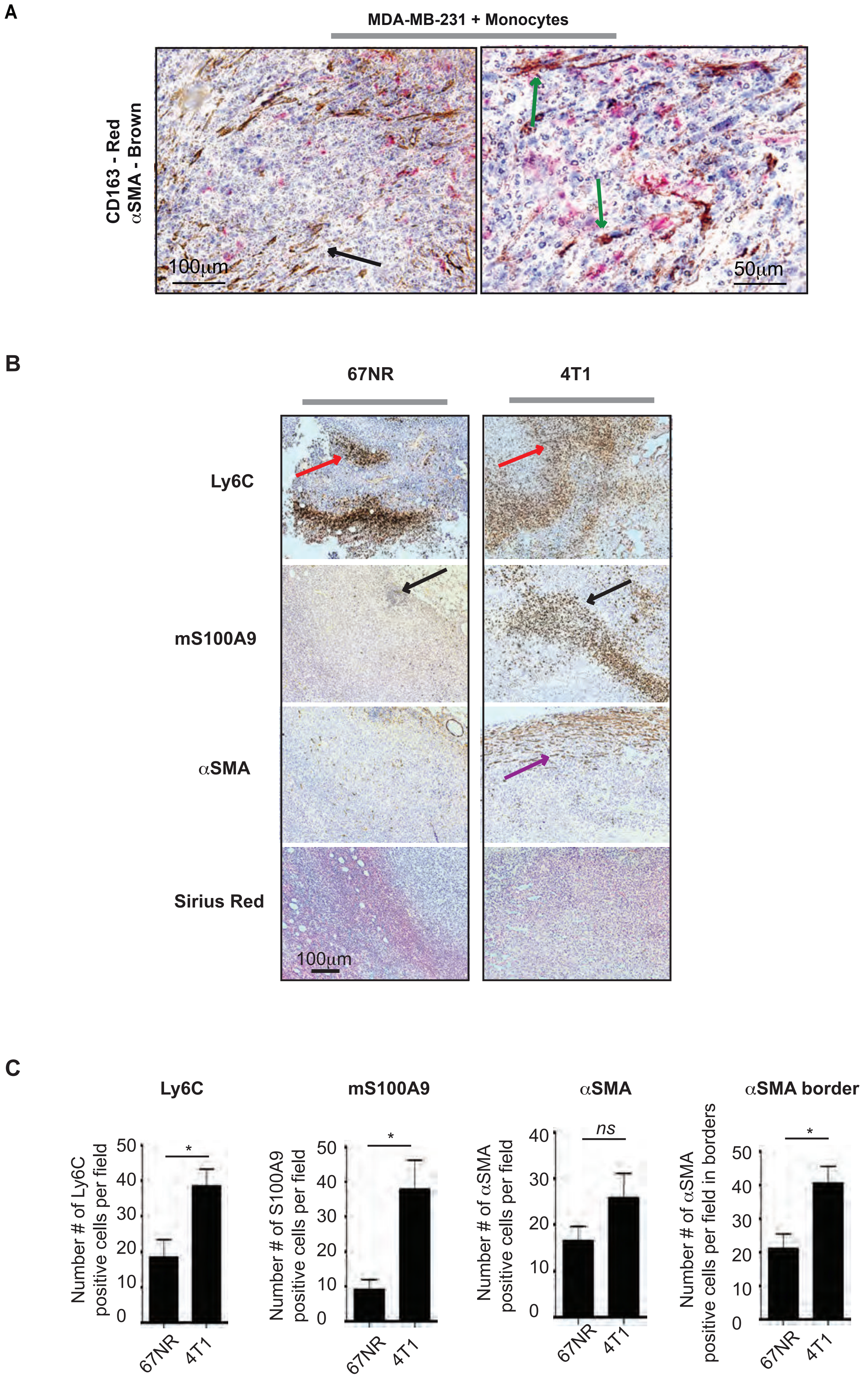
A
MDA-MB-231 + Monocytes
CD163 - Red
αSMA - Brown
100μm
50μm
B
67NR
4T1
Ly6C
mS100A9
αSMA
Sirius Red
100μm
C
Ly6C
mS100A9
αSMA
αSMA border
Number # of Ly6C positive cells per field
Number # of S100A9 positive cells per field
Number # of αSMA positive cells per field
Number # of αSMA positive cells per field in borders
ns
*
67NR
4T1
0
10
20
30
40
50

Supplementary Figure 5. *Immunohistochemistry of xenografts and syngeneic tumors*

(A) Double immunohistochemical staining of CD163 and αSMA in the MDA-MB-231 / monocytes xenografts. Black arrows show staining with only αSMA and green arrows show double staining.

(B) Syngeneic mouse tumors consisting of luminal A (67NR) or TN (4T1.13) breast cancer cells transplanted into BALB/c mice. The TN 4T1.13 grafts express more myeloid-related (Ly6C), immunosuppressive (S100A9) and activated fibroblast (αSMA) markers than the luminal 67NR tumors. Sections from the tumors were stained with myeloid (Ly6C and S100A9) and the activated fibroblast marker αSMA. Immunohistochemistry was completed using the indicated antibodies. All histological sections were counterstained with HE.

(C) The histograms show the mean value for each IHC score with statistical analysis. For scoring five fields were counted per staining. *=$p<0.05$ Mann-Whitney U-test. Error bars indicate SEM.

Fig 6

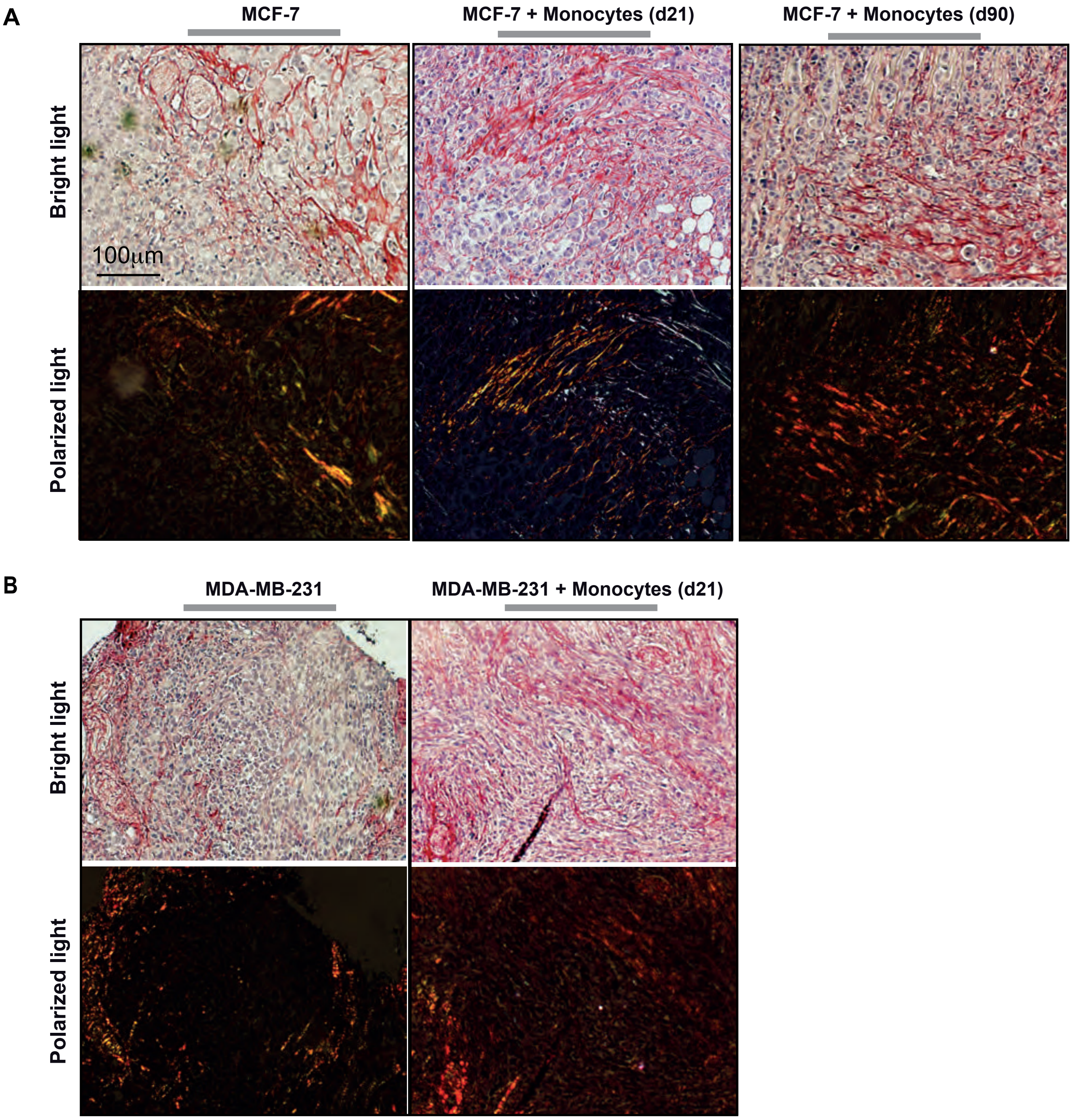
A
MCF-7
MCF-7 + Monocytes (d21)
MCF-7 + Monocytes (d90)
Bright light
Polarized light
100μm
B
MDA-MB-231
MDA-MB-231 + Monocytes (d21)
Bright light
Polarized light

Supplementary Figure 6. *Sirius Red staining of xenografts*

(A) Sirius Red staining of the stroma (top row; bright light microscope; red) and classical collagen bundles (bottom row; polarized light microscope; red/green/yellowish stain) on; MCF-7 or MCF-7 / monocytes on day 21 and day 90, as indicated.

(B) Sirius Red staining of the stroma (top row; bright light microscope; red) and classical collagen bundles (bottom row; polarized light microscope; red/green yellowish stain) on; MDA-MB-231 or MDA-MB-231 / monocytes xenografts. (N=5 for each group) on day 21.

Fig 7

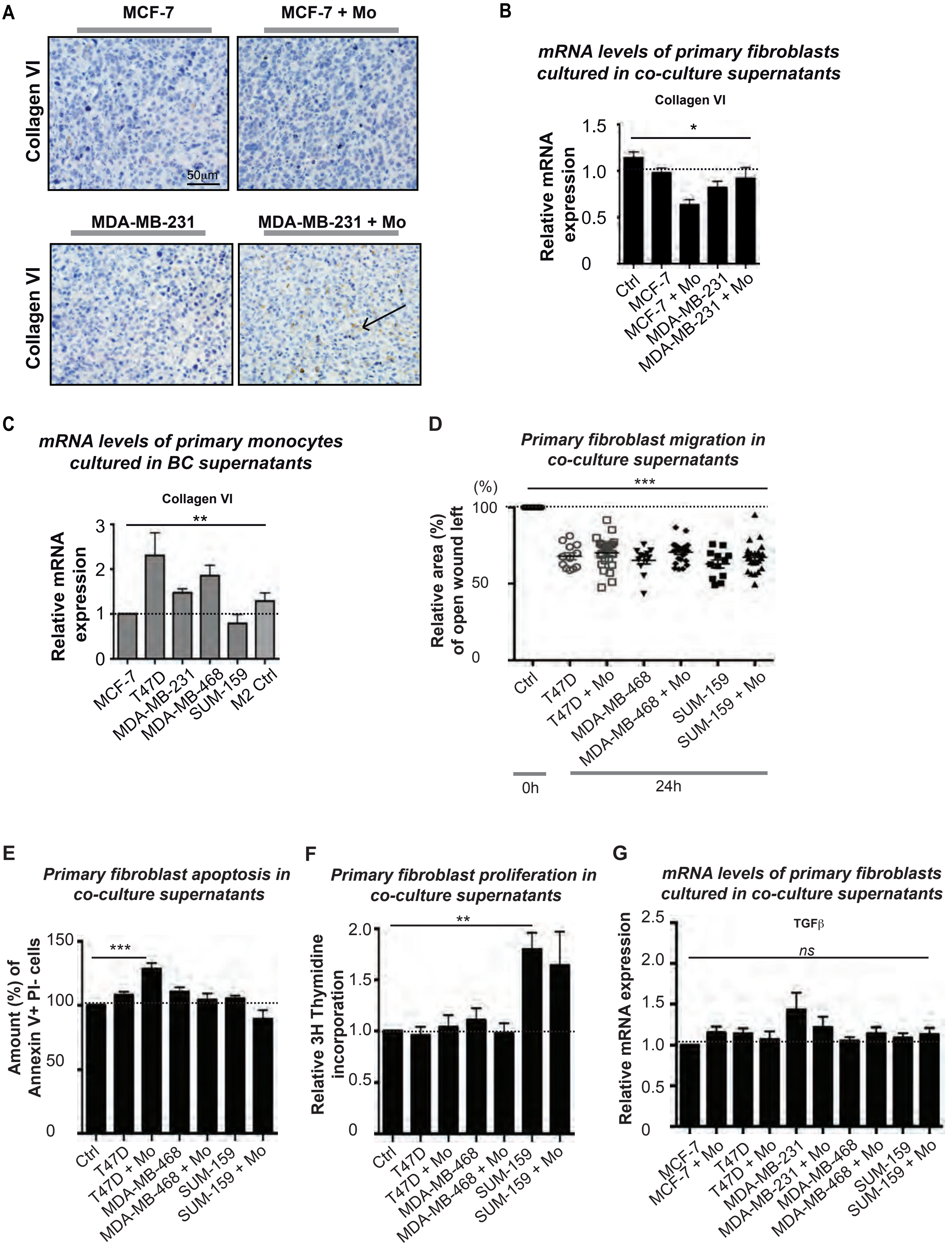
A
MCF-7
MCF-7 + Mo
Collagen VI
50μm
MDA-MB-231
MDA-MB-231 + Mo
Collagen VI
B
mRNA levels of primary fibroblasts cultured in co-culture supernatants
Collagen VI
Relative mRNA expression
1.5
1.0
0.5
0
*
Ctrl
MCF-7
MCF-7 + Mo
MDA-MB-231
MDA-MB-231 + Mo
C
mRNA levels of primary monocytes cultured in BC supernatants
Collagen VI
Relative mRNA expression
3
2
1
0
**
MCF-7
T47D
MDA-MB-231
MDA-MB-468
SUM-159
M2 Ctrl
D
Primary fibroblast migration in co-culture supernatants
(%)
***
Relative area (%) of open wound left
100
50
0
Ctrl
T47D
T47D + Mo
MDA-MB-468
MDA-MB-468 + Mo
SUM-159
SUM-159 + Mo
0h
24h
E
Primary fibroblast apoptosis in co-culture supernatants
Amount (%) of Annexin V+ PI- cells
150
100
50
0
***
Ctrl
T47D
T47D + Mo
MDA-MB-468
MDA-MB-468 + Mo
SUM-159
SUM-159 + Mo
F
Primary fibroblast proliferation in co-culture supernatants
Relative 3H Thymidine incorporation
2.0
1.5
1.0
0.5
0
**
Ctrl
T47D
T47D + Mo
MDA-MB-468
MDA-MB-468 + Mo
SUM-159
SUM-159 + Mo
G
mRNA levels of primary fibroblasts cultured in co-culture supernatants
TGFβ
ns
Relative mRNA expression
2.5
2.0
1.5
1.0
0.5
0
MCF-7
MCF-7 + Mo
T47D
T47D + Mo
MDA-MB-231
MDA-MB-231 + Mo
MDA-MB-468
MDA-MB-468 + Mo
SUM-159
SUM-159 + Mo

Supplementary Figure 7. *Collagen IV expression in TNBC xenografts and cultures*

Collagen VI is expressed by myeloid cells in a triple-negative breast tumor context.

(A) Xenografts of luminal A MCF-7 or TN MDA-MB-231 breast cancer cells, alone (left) or with primary human monocytes (Mo; right) in NSG-mice. Immunohistochemistry was performed using antibodies to collagen VI. Black arrow indicate collagen VI expression in TN / monocyte grafts. All histological sections were counterstained with HE.

(B) Collagen VI mRNA expression levels measured by RT-QPCR in primary mouse fibroblasts grown in breast cancer / monocyte co-culture supernatants. *=$p<0.05$ ANOVA. N=4.

(C) Collagen VI mRNA expression levels measured by RT-QPCR in human myeloid cells cultured in breast cancer supernatants. Primary human M2 macrophages = positive control*=$p<0.05$ ANOVA. N=4. Error bars indicate SEM.

(D) Scratch wound assays showing mouse primary fibroblast migration in supernatants derived from co-cultures of human primary monocytes (Mo) and luminal A (T47D) or TN (MDA-MB-468 and SUM-159) breast cancer cells. ***=$p<0.001$ ANOVA non-parametric Kruskal Wallis test. N=20.

(E) Survival analysis of mouse primary fibroblasts grown in supernatants derived from co-cultures of human primary monocytes and luminal A (T47D) or TN (MDA-MB-468 and SUM-159) breast cancer cells. Annexin V staining was performed to analyze the percentage apoptotic cells. ***=$p<0.001$ ANOVA Dunn's multiple comparison test. N=10.

(F) Proliferation of mouse primary human fibroblasts grown in supernatants derived from co-cultures of human primary monocytes and luminal A (T47D) or TN (MDA-MB-468 and SUM-159) breast cancer cells, measured using a thymidine incorporation proliferation assay. **=$p<0.01$ ANOVA Dunn's multiple comparison test. N=14.

(G) mRNA expression levels of TGFβ in mouse primary fibroblasts cultured in supernatants derived from co-cultures of human primary monocytes and luminal A (MCF-7 and T47D) or TN (MDA-MB-231, MDA-MB-468 and SUM-159) breast cancer cells, assessed by RT-QPCR analysis. ns=non-significant ANOVA Dunn's multiple comparison test. N=4-8.

Supplementary Table 1.

Histological and immunohistochemistry scores of xenografts consisting of luminal A MCF-7 or triple-negative (TN) MDA-MB-231 (231) breast cancer cells, alone or co-transplanted with primary human monocytes (Mo), in NSG mice

	MCF-7 (1x10^{6}cells)	MCF-7+Mo (1x10^6 + 1x10^6)	231 (1x10^6)	231+Mo (1x10^6 + 1x10^6)
Size (mm) [1]	3	X	1	2
	3+2[2]	2	1.5	1
	3+4[2]	2.5	4	1
	3	2+1[2]	5	2
	4	2	3	4
CD11b (0-2) [1]	0	X	0	1
	0	1	0	1
	0	*1*	0	1
	0	0	0	2
	0	1	0	2
CD68 (0-3) [3]	0	X	0	1
	0	0	0	1
	0	0	0	0
	0	0	0	1
	0	0	0	1
CD163 (0-3) [1]	0	X	0	1
	0	0	0	1
	0	0	0	1
	0	0	0	3
	0	1	0	3
S100A9 (0-3) [1]	0	X	0	2
	0	0	0	2
	0	0	0	2
	0	0	0	2
	0	0	0	2
Vimentin (0-2) [1]	0	X	2	2
	0	1	2	2
	0	1	2	2
	0	1	2	2
	0	1	2	2
αSMA (0-4) [1]	1	X	2	3
	1	2	3	3
	1	1	2	3
	1	2	2	3
	2	1	2	4
β2-microglobulin (%) [1]	25	X	50-75	50-75
	10	25-50	25	>75
	10	25-50	10	>75
	25-50	75	10	50
	25-50	50-75	25	75
PDGFRβ (0-1) [1]	1	X	1	1
	1	1	1	1
	1	1	1	1
	1	1	1	1
	1	1	1	1
Sirius Red (%) [1]	50	X	25-50	75-100
	50	50-75	25	25-50
	50	50-75	25	50-75
	50	50-75	25-50	10-25
	50	75-100	25	10-25
Sirius Red (%) PL [1]	10	X	0-10	25
	25	25	0-10	0-10
	25	50	0-10	10
	25	50	0-10	0-10
	25-50	75-100	0-10	0-10

Collagen VI (0-1) [4]	0	X	0	1
	0	0	0	1
	0	0	0	1
	0	0	0	1
	0	0	0	1
CD34 (0-2) [1]	2	X	2	2
	0	1	2	2
	0	0	2	0
	1-2	1-2	2	2
	1	2	2	2

[1] For statistics see Fig. 1, Fig. 2, Fig. 3 and Supplementary Fig. 1.

[2] Two tumors

[3] Statistics for CD68 not shown in Figures: (MDA-MB-231 + Mo) CD68 expression as compared to (MCF-7 + Mo); *$p<0.05$ (t-test)

[4] Statistics for Collagen VI not shown in Figures: (MDA-MB-231 + Mo) Collagen VI expression as compared to (MCF-7 + Mo); **$p<0.01$ (t-test)

[x] No tumor

PL = polarized light

Supplementary Table 2.

Histological and immunohistochemistry scores of xenografts consisting of luminal A T47D or triple-negative (TN) SUM-159 breast cancer cells, alone or co-transplanted with primary human monocytes (Mo), in NSG mice

	T47D (5x10[6]cells)	T47D+Mo (5x10[6] + 1x10[6])	SUM-159 (1x10[6])	SUM-159+Mo (1x10[6] + 1x10[6])
Size (mm)[1]	4	1.5	3	5
	3	1.5	5	7
	X	1	3	6.5
	4	2	2	5
	4	2	3	5
CD11b (0-2) [1]	0	0	0	1
	0	0	0	2
	X	0	0	2
	0	1	0	2
	0	1	0	1
CD163 (0-3) [1]	0	0	0	3
	0	0	0	3
	X	0	0	3
	0	0	0	3
	0	0	0	1
S100A9 (0-3) [1]	0	1	1	2
	1	1	0	3
	X	1	1	3
	0	1	1	3
	1	1	0	2
αSMA (0-4) [1]	1	0	2	4
	1	1	1	4
	X	2	2	4
	0	0	2	4
	1	1	3	4
Sirius Red (%)[1]	67	75	25	50
	50	75	75-100	25-50
	X	75	10	50
	25	50	75-100	50
	75	50	25	25-50
Sirius Red (%) PL[1]	50	50	10-25	10-25
	25-50	75	75	10
	X	75	10	25
	25-50	50	25	10-25
	50	75	0-10	10
Collagen VI (0-1)[2]	0	0	0	1
	0	0	1	1
	X	0	0	1
	0	0	1	1
	0	0	1	1

[1] For statistics see Supplementary Fig. 1 and Fig. 3.

[2] Statistics for Collagen VI not shown in Figures: (T47D + Mo) Collagen VI expression as compared to (SUM-159 + Mo); **p<0.01 (t-test)

[x] No tumor

PL = polarized light

Supplementary Table 3. Gene expression of ACTA2, Ly6C, S100A9 and CXCL16 in mouse TNBC 4T1.2 tumors compared to mouse luminal 67NR tumors.

Gene	Gene Name	Whole tumor gene array data (4T1.2 vs 67NR) (Fold change)	Adjusted P value
ACTA2	Alpha smooth muscle actin	3.51	3.34E-03
Ly6C	Lymphocyte antigen 6 complex	1.89	0.788
S100A9	S100 Calcium binding protein A9	7.03	1.75E-04[1]
CXCL16	Chemokine (C-X-C-Motif) Ligand 16	2.74	1.73E-02

[1] Already published data [2]

Supplementary Table 4.

Antibodies used for immunohistochemistry (specificity[1]; clone; dilution; distributor)

anti-CXCL16 (specific for human; ab101404 dilution 1:100; Abcam)

anti-CD11b (specific for human; clone #EP1345Y dilution 1:100; Abcam)

anti-CD163 (specific for human; clone #10D6 dilution 1:250; Novocastra)

anti-CD68 (specific for human; dilution 1:1500; DAKO)

anti-vimentin (clone #V9 dilution 1:1000; Dako)

anti-αSMA (recognizes both mouse and human origin; clone #1A4 dilution 1:1000; Dako)

anti-human S100A9 (specific for human; calgranulin B clone #H90 dilution 1:2000; Santa Cruz)

anti-mouse S100A9 (specific for mouse; ab105472 dilution 1:100; Abcam)

anti-β2microglobulin (specific for mouse; sc-8361 dilution 1:100; Santa Cruz)

anti-PDGFRβ (clone #3169 dilution 1:100; Cell Signaling)

anti-Collagen VI (recognizes both mouse and human origin; clone #H-200 dilution 1:250; Santa Cruz)

anti-HLA-ABC (specific for human; Ab70328 dilution 1:2000; Abcam)

anti-CD34 (specific for mouse; clone #MEC14.7 dilution 1:800; Santa Cruz)

anti-F4/80 (specific for mouse; clone #Cl:A3-1 dilution 1:2000; Abcam)

anti-DC-LAMP (specific for human; CD208; clone #101E1.01 dilution 1:1000; Dendritics)

anti-CD90 (specific for human; clone #EPR3132 dilution 1:250; Abcam)

anti-Ly6C (specific for mouse; ab15627 dilution 1:100; Abcam)

anti-Gr1 (specific for mouse; clone #RB6-8C5; Nordic Biosite)

---

[1] *Specificity (mouse vs human) tested for all antibodies*

Supplementary Table 5. Primers used in Quantitative real-time PCR

GENES	FORWARD	REVERSE
Mouse ACTB	CTCTGGCTCCTAGCACCATGAAGA	CATGATGCTTGATCACATGTCTCG
Mouse HPRT	CAAGCTTGCTGGTGAAAAGGAC	GTCAAGGGCATATCCTACAACAAA
Mouse GAPDH	TGCACCACCAACTGCTTAG	GATGCAGGGATGATGTTC
Mouse alpha-SMA	ACTGGGACGACATGGAAAAG	GTTCAGTGGTGCCTCTGTCA
Mouse TGF-B	GGATACCAACTATTGCTTCAGCTCC	AGGCTCCAAATATAGGGGCAGGGTC
Mouse FAP	ACTGGGTGTATATGAAGTTGAGGAC	TTCTTCATCAATGAAACCCATTT
Mouse CXCL16	product number: 100-25636, Bio-Rad	
Mouse COL6A1	CCACAGGGTGACCAAGGAAG	ACCTCGGTATCCTTTAGGTCCAA
Human ACTB	CTGGAACGGTGAAGGTGACA	AAGGGACTTCCTGTAACAATGCA
Human GAPDH	TGCACCACCAACTGCTTAGC	GGCATGGACTGTGGTCATGAG
Human SDHA	TGGGAACAAGAGGGCATCTG	CCACCACTGCATCAAATTCATG
Human YWHAZ	ACTTTTGGTACATTGTGGCTTCAA	CCGCCAGGACAAACCAGTAT
Human UBC	ATTTGGGTCGCGGTTCTTG	TGCCTTGACATTCTCGATGGT
Human COL6A1	ACCGACTGCGCTATCAAGAA	TCGGTCACCACAATCAGGTA

Supplementary References

1 Bergenfelz, C. *et al.* S100A9 expressed in ER(-)PgR(-) breast cancers induces inflammatory cytokines and is associated with an impaired overall survival. *Br J Cancer* **113**, 1234-1243, doi:10.1038/bjc.2015.346 (2015).

2 Johnstone, C. N. *et al.* Functional and molecular characterisation of EO771.LMB tumours, a new C57BL/6-mouse-derived model of spontaneously metastatic mammary cancer. *Dis Model Mech* **8**, 237-251, doi:10.1242/dmm.017830 (2015).
